# Delivery of AgNP led to more pronounced local retention in the subcutaneous versus intraperitoneal location with limited uptake and toxic effects in rats

**DOI:** 10.1111/vsu.70087

**Published:** 2026-03-12

**Authors:** Marije Risselada, Makensie L. Anderson, Miriam G. Bates, Abigail Cox, Robyn McCain, Kristen Messenger

**Affiliations:** ^1^ Department of Veterinary Clinical Sciences Purdue University West Lafayette Indiana USA; ^2^ Arizona Regional Intensive Care, Specialty and Emergency Veterinary Center Queen Creek Arizona USA; ^3^ Department of Comparative Pathobiology Purdue University West Lafayette Indiana USA; ^4^ Center for Clinical Translational Research College of Veterinary Medicine Purdue University West Lafayette Indiana USA; ^5^ Department of Biomedical Sciences and Pathobiology Virginia‐Maryland College of Veterinary Medicine Blacksburg Virginia USA

## Abstract

**Objective:**

To determine adverse effects of intraperitoneally (IP) or subcutaneously (SC) delivered silver nanoparticles (AgNP) in poloxamer 407 (P407; AgNP‐P407) and local retention and systemic uptake in rats.

**Study design:**

Controlled randomized trial.

**Animals:**

A total of 25 rats.

**Methods:**

A total of 10 rats were randomly assigned in Phase 1 to a SC or IP surgical delivery group (5 rats/group) to receive 0.01 mg AgNP/rat in P407 (1:2 volume ratio). A total of 15 rats (5/group) were used in Phase 2 to receive IP: gel only (1.0 mL), AgNP‐P407 (0.01 mg AgNP in 1.0 mL P407) or AgNP (0.01 mg in aqueous buffer). Incisions were assessed daily. Euthanasia was performed on day 7. The SC surgery sites and organs were harvested and evaluated histologically with scores reported. Plasma was obtained and batch analyzed together with organs for Ag content using mass spectroscopy.

**Results:**

All rats survived the duration of the study. Minimal local histologic toxicity was seen after SC delivery. Plasma Ag peaked earlier for IP than SC delivery (*p* = .08) and for Ag delivered IP without gel (*p* = .04). Organ Ag uptake was minimal; macrophage aggregation was identified in the spleen and lymph node consistently. Rats that received P407 had increased cholesterol, whereas changes in total protein, albumin, glucose, amylase and lipase were seen for all groups.

**Conclusion:**

No adverse events were noted after use of AgNP‐P407, while Ag uptake was delayed.

**Clinical relevance:**

Use of AgNP‐P407 may be possible without systemic effects and prolonged local presence, but further testing is needed.

## 1 INTRODUCTION

Bacterial infections can increase patient risk, treatment cost, and prolong hospitalization stays. Development of antimicrobial resistant infection during treatment or acquiring a hospital multi‐drug resistant (MDR) infection further increases these risks.^1^, ^2^ Multi‐drug resistance has increased over the past decades in both human and veterinary medicine.^3^, ^4^, ^5^, ^6^ Inability to treat infections is having an increasingly large impact on healthcare. The use of agents with minimal documented bacterial resistance is needed and has gained renewed interest. One such agent is nanoparticulate silver (AgNP). One hypothesis on the mechanism of action is that silver ions act directly on bacterial membranes thereby creating permanent damage.^7^ This bactericidal effect in 10 nm AgNPs is by combined ion‐particle action, as opposed to larger sized AgNPs where bacteria do not internalize the particles.^8^ Efficacy of AgNP has been shown prior against non‐multi‐drug resistant (MDR) *Staphylococcus aureus*,^9^ as well as MDR *Staphylococcus aureus*(MRSA),^10^ *Pseudomonas aeruginosa* ^11^, ^12^ and *Escherichia coli*.^12^ Minimum inhibitory concentrations (MICs) for AgNPs on bacterial isolates of 2.5–5 μg/mL (human isolates of MRSA)^3^ 4.9 μg/mL (MRSA)^10^ and 1 μg/mL (*P. aeruginosa* in milk samples from mastitis‐infected goats)^11^ have been reported. Irreversible inhibition of growth at a very short (1‐h duration) exposure was found for *P. aeruginosa* and *E. coli* at 40 μM (43 mg/mL) and 320 μM (35 mg/L) respectively,^12^ with efficacy ranges most likely due to the formulation and delivery method of the NPs.

Silver (Ag) is used currently clinically in the form of coated implants^13^ and is heavily used in open wound management.^14^, ^15^ Coated implants are aimed at preventing infection, especially in the face of a contaminated environment,^13^ whereas wound dressings are used therapeutically in chronic wounds.^14^, ^15^ However, currently no products are available aimed at providing ongoing local therapy after wound closure, or to provide continued treatment for abscesses in organs or complicated locations, for example pancreatic or prostatic abscesses, or a body wall abscess.

Poloxamers consist of three distinct blocks: Poly(ethylene glycol)‐block‐poly(propylene glycol)‐block‐poly(ethylene glycol) (PEO/PPO/PEO). Their properties rely on the block length, with their nomenclature reflecting their state at room temperature with P indicating “paste”, and the numbers reflecting the molecular weights.^16^ Poloxamer 407 is classified as a thermo reversible gel: the gel will return to a solution if the temperature change is reversed.^17^ It gelifies at body temperature or higher temperatures and returns to a liquid state at room temperature or below.^18^ This process can be repeated to help mix active substances into the gel, aid delivery of the gel‐active substance into tissues via a red rubber catheter,^19^ ultrasound‐guided injection,^20^ or applied as a gel in a closed surgery site or onto a wound bed.^20^ Its elution properties for several drugs and active substances have been studied for P407 alone as well as for modifications, such as the addition of hyaluronic acid.^21^ Poloxamer 407 has been used as a sustained release carrier to deliver various drugs, such as:chemotherapeutics,^22^, ^23^, ^24^ antibiotics,^16^, ^19^, ^25^, ^26^ analgesics^18^, ^21^, ^27^ and antifungals^18^ as well as AgNP in a wound application.^28^ Poloxamer 407 on its own did not incite any inflammatory response^19^, ^22^ and it was found to have an inhibitory efficacy against 10^8^ *E. coli* and 10^5^ MRSP when applied simultaneously with bacterial inoculation.^29^ One mechanism of action of P407 itself is destabilizing bacterial cell membranes by interaction of the hydrophobic segments with lipid bilayers.^16^, ^30^ This property could enhance its effectiveness in the face of antimicrobial resistance and led to our choice to further investigate its use as a vehicle to deliver antimicrobials locally. In vitro, nanoparticulate Ag delivered in P407 (AgNP‐P407) as a sustained release carrier had superior antimicrobial efficacy compared to AgNP delivered in a hemostatic gelatin sponge or CaSO_4_ beads for both *E. coli* and methicillin‐resistant *S. pseudintermedius* (MRSP).^29^ However, the antimicrobial effect at the dose of silver investigated might be better suited for use as an adjunctive treatment or for use in a contaminated wound (surgically classified as <10^5^ organisms/g tissue) than for sole treatment, unless a higher dose of AgNP can be mixed with P407.

Our aims were to determine (1) adverse effects after intraperitoneal (IP) or subcutaneous (SC) surgical delivery of AgNP‐P407 in rats and (2) distribution of Ag to include systemic uptake in circulation, organ concentrations, and local retention at the site of delivery. We hypothesized that (1) no local toxic effects would be seen after SC or IP surgical delivery of AgNP‐P407 in rats and (2) little systemic absorption of AgNP and no adverse organ pathology would be noted after SC or IP delivery while maintaining high local concentrations.

## 2 MATERIALS AND METHODS

A commercial 0.02 mg/mL spherical, 10 nm diameter, silver nanoparticle (AgNP)dispersion in aqueous buffer with sodium‐citrate as stabilizer was used as stock solution (Sigma Aldrich; Massachusetts). The stock was shielded from light and refrigerated (40°F) while not in use and filtered using a 0.2 μm filter (Corning; Arizona) immediately prior to preparation of individual doses. Poloxamer 407 (PCCA poloxamer 407 NF gel 30%, PCCA, Houston, Texas) was distributed into a 100 mL multi‐dose injection vial, steam sterilized within 7 days prior to the study start date, shielded from light and refrigerated until use.

Each individual dose of AgNP was 0.01 mg AgNP/rat, either delivered as AgNP only, or with P407 in a 2:1P407 to AgNP ratio (AgNP‐P407). The AgNP only dose was 0.5 mL of the 0.02 mg/mL stock solution, the AgNP‐P407 dose was 0.5 mL of the 0.02 mg/mLAgNP stock solution in 1.0 mL P407. Individual doses for each rat were drawn up sterilely in individual 3 m Lluer slip syringes (Thermo Scientific, Rockwood, Tennessee) the morning of delivery and kept refrigerated and shielded from light until use. The AgNP‐P407 was mixed by injecting the AgNP into the syringe with the volume of drawn up P407 in liquid state. This syringe was then capped, agitated and kept refrigerated until use. At the time of use, the prepared doses were kept at room temperature and allowed to gelatinize prior to implantation.

### 2.1 Rats

A total of 25 intact male Sprague Dawley rats (Envigo, Indianapolis, Indiana) were used after obtaining institutional approval (IACUC#: 191000195). Rats were housed in agreement with the standard of care in the Guide for the Care and Use of Laboratory Animals.^31^

A total of 10 rats were used in Phase 1 and 15 rats in Phase 2 (Table 1). Delivery routes of AgNP‐P407 (0.5 mL of 0.02 mg/mL AgNP in 1.0 mL P407) were compared in Phase 1: one group with subcutaneous (SC) delivery (*n* = 5) and one group with surgical intraperitoneal (IP) delivery (*n* = 5).The rats with SC delivery underwent a control SC surgery for histologic comparison in which only P407 was delivered. Three compositions were compared in Phase 2 (*n* = 5 each, all delivered surgically IP): P407 only (1.0 mL 30% P407), AgNP‐P407 (0.5 mL of 0.02 mg/mLAgNP in 1.0 mL P407) or AgNP only (0.5 mL of 0.02 mg/mLAgNP).

**TABLE 1:** Summary of treatments and diagnostic sampling.

	Phase 1: route		Phase 2: formulation delivered IP		
	IP	SC	AgNP	AgNP‐P407	P407
**Rats**	5	5	5	5	5
**Dose & volume administered**					
Total dose AgNP	0.01 mg	0.01 mg	0.01 mg	0.01 mg	
Volume AgNP delivered with P407	0.5 mL	0.5 mL	0.5 mL	0.5 mL	
Volume P407 delivered with AgNP	1.0 mL	1.0 mL		1.0 mL	1.0 mL
Volume P407 delivered in Sham Surgery		1.0 mL			
**CBC/chemistry**					
Preoperative			Yes	Yes	Yes
Postoperative	Yes	Yes	Yes	Yes	Yes
**Samples Ag**					
Plasma	Yes	Yes	Yes	Yes	Yes
Surgery site/Incision		Yes	Yes	Yes	Yes
Kidney, liver, lung	Yes	Yes			
Kidney, liver, lung, pancreas, spleen					
**Wound scoring**	Yes	Yes	Yes	Yes	Yes
**Histopathology**					
*Assessment*					
Surgery site	Yes				
Sham surgery site	Yes	n/a	n/a	n/a	n/a
Heart, liver, spleen, lung and kidneys	Yes	Yes			
Thymus, heart, lung, liver, spleen, pancreas, duodenum, abdominal lymph nodes and kidneys			Yes	Yes	Yes
*Scoring of tissue inflammation*					
Surgery site	Yes		Yes	Yes	Yes
Sham surgery site	Yes	n/a	n/a	n/a	n/a

*Note*: The route of administration differed in Phase 1, whereas the administration route was kept constant in Phase 2 but the composition delivered differed. Unaltered AgNP stock was delivered in the aqueous buffer of the stock solution.

### 2.2 Anesthesia protocols and surgical procedures

Rats were anesthetized in an induction chamber at 0.5%–5% isoflurane mixed with oxygen and maintained during surgery via mask with isoflurane (0.5%–5%) in oxygen as needed with the upper limit of 5% only used initially, and maintenance during surgery at 1%–2%. This protocol was used for anesthesia episodes for collection as well as for surgery. Peri‐ and postoperative analgesia was provided by a 0.65 mg/kg subcutaneous injection of buprenorphine extended release (Ethiqua XR, Fidelis Animal Health, North Brunswick, New Jersey). The location of the injection was on the side of the rat opposite to the surgical incision (i.e., ventral for the SC dorsal incisions, and dorsal for the abdominal incision). All rats were visually inspected during recovery until they were able to ambulate.

### 2.3 Delivery route (Phase 1)

#### 2.3.1 Surgical IP delivery

The surgical IP delivery group had one procedure performed: a 5 mm skin incision over the ventral midline was made using a #15 blade (McKesson, Richmond, Virginia), the linea alba was stabilized with Brown Adson forceps, and a stab incision was made into the abdomen using the same #15 blade. A cruciate suture (4–0 poliglecaprone 25; Monocryl, Ethicon, Somerville, New Jersey) was preplaced in the linea prior to inserting the hub of the luer slip syringe intra‐abdominally. The suture was tensioned and the abdominal wall elevated while injecting the volume of AgNP‐P407. The cruciate suture was then immediately tied while maintaining tension to avoid leakage. The wound was closed by closing the skin with a buried cruciate pattern using 4–0 poliglecaprone 25. Glue was applied externally (GLU ture, World Precision Instruments, Sarasota, Florida) to decrease the risk of dehiscence due to self‐trauma.

#### 2.3.2 Surgical subcutaneous delivery

The rats in the SC delivery group had two procedures performed under the same anesthesia: (1) the implantation of AgNP‐P407 and (2) a control implantation site with P407 only. The implantation of AgNP‐P407 was performed in the dorsal lumbar subcutaneous area. Poloxamer 407 was implanted in the dorsal cervical subcutaneous area. For each, a 5 mm skin incision was made using a #15 blade. A subcutaneous pocket was created by blunt dissection using tenotomy scissors. A buried cruciate suture (4–0 poliglecaprone 25) was preplaced in the skin, and either P407 or AgNP‐P407 doses were injected into the SC space while tensioning and tightening the suture as described for the IP delivery. Additional glue was placed externally to complete skin closure.

### 2.4 Delivery composition (Phase 2)

A total of 15 rats were allocated to three groups (*n* = 5 each) with IP delivery only of different compositions: P407, AgNP‐P407, or AgNP. Anesthesia and IP delivery in Phase 2 were performed in the same manner as the IP delivery in Phase 1.

### 2.5 Postoperative data collection

Rats were maintained for 7 days postoperatively to monitor surgery sites daily for dehiscence, discharge, redness and swelling using parameters reported previously.^32^ Bodyweight (BW) in grams as well as animal activity and behavior were evaluated daily. This included voluntary food intake, body condition, grooming behavior, and activity levels.

Blood for a complete blood count (CBC) and chemistry panel was obtained at the time of euthanasia for all rats (Phases 1 and 2) as well as prior to the study in Phase 2. When samples were insufficient for both panels, the chemistry panel was prioritized. Additional blood samples (0.3 mL per time point) for Ag analysis were taken at 6 h, 12 h, day 1, day 2, and day 4. Venipuncture was performed from the tail vein (6 h), submandibular vein (12 h) and tail vein for the remainder. Euthanasia was planned for day 7 in all rats. One final large volume blood for CBC, chemistry as well as Ag analysis was obtained after induction of deep general anesthesia using isoflurane (5%) in oxygen and prior to establishing pneumothorax.

### 2.6 Quantitative silver analysis

All blood samples were spun immediately, and the resulting plasma snap frozen and stored at −80°C until batch analysis at the UNC nanomedicine characterization core facility according to previously published methodology, and internally validated protocols.^33^ Tissue samples were collected on day 7 and immediately frozen at −80°C: the SC delivery site with AgNP‐P407 (but not abdominal incision), kidney, liver, and lung for Phase 1. In Phase 2, the abdominal incision, kidney, liver, lung, pancreas, and spleen were sampled. All plasma and tissue samples were digested overnight at 70°C in a 2:1 mixture of 70% H_2_NO_3_ and H_2_O_2_. Analysis was performed utilizing ICP‐MS (Perkin Elmer NexION 300D) to determine the concentration of Ag within each sample expressed as Ag/g tissue. The short‐term precision was less than 3% relative SD, and the long‐term stability was <4% relative SD over 4 h. Isotope‐ratio precision was less than 0.08% relative SD. The Ag detection limit was 0.001 ng/mL, the quantification limit (LOQ) at 0.002 ng/mL and all samples below this limit of quantification (BLOQ) were recorded as 0 ng/mL.

### 2.7 Histologic assessment

Following euthanasia, histology samples of the incision sites for the rats with SC delivery in Phase 1, as well as the incision sites for the abdominal approach in all rats during Phase 2 were collected. Additionally, the following organs were collected to assess possible organ pathology: heart, liver, spleen, lung and kidneys (Phase 1); thymus, heart, lung, liver, spleen, pancreas, duodenum, abdominal lymph nodes and kidneys (Phase 2). Any macroscopically visible lesions in the abdominal cavity were collected. All tissue samples were fixed in 10% neutral‐buffered formalin for routine histologic processing. In addition to hematoxylin and eosin (H&E) stains, immunohistochemistry (IHC) was performed on splenic tissue of rats from Phase 2 to better delineate and distinguish adipocytes from macrophages, using CD68 labeling.

The SC implantation site and sham surgery site in Phase 1 and abdominal incision sites in Phase 2 were assessed histologically by a boarded veterinary pathologist to evaluate tissue inflammation and/or reaction using prior established criteria.^22^, ^32^ Microscopic lesions (inflammation and leukocyte types, necrosis, edema, hemorrhage, fibrin, fibrosis) and their severity were semi‐quantitatively scored as follows: 0 = no lesions; 1 = minimal; 2 = mild; 3 = moderate; 4 = marked.

### 2.8 Pharmacokinetics

Plasma concentration data was analyzed using noncompartmental pharmacokinetic methods. All analyses were performed using Phoenix WinNonlin version 8.2 (Certara). The maximum plasma concentration (Cmax) and time to maximum plasma concentration (Tmax) were calculated directly from the data. The AUC from time 0 to the last time point, and time 0 to infinity were estimated using the linear log trapezoidal method (linear to Cmax, log to the last time point). Standard non‐compartmental equations were used to obtain other reported parameters.^34^ The data are summarized using descriptive statistics (median, range).

### 2.9 Statistical analysis

Pharmacokinetic parameters were compared to each other for Phase 1 and Phase 2 studies (i.e., Phase 1 SC was compared to Phase 1 IP). Pharmacokinetic parameters were tested for normality using the Shapiro–Wilk test. Normally distributed data were compared using a Welch's *t*‐test, and nonparametric data were compared using a Mann–Whitney test, and *p* ≤ .05 was considered significant. All statistical analyses of the pharmacokinetic data were performed using GraphPad Prism version 10.6 for Windows (GraphPad Software, Boston, Massachusetts, www.graphpad.com). The Ag data was presented in a descriptive manner. A Shapiro Wilk test (www.statskingdom.com) was performed on the histologic scores for the SC control surgery site and SC implantation site. Non‐normally distributed data were compared with a Mann Whitney U test (www.statskingdom.com) and normally distributed data were compared using a paired *t*‐test (using Excel Microsoft Excel, Microsoft 365, Microsoft corporation, Redmond, Washington). Significance level of *p* was set at <.05.

## 3 RESULTS

### 3.1 Rats

All rats survived to the planned end point (7 days). The mean BW of all rats was 293.4 ± 11.6 g. Mean rat BW was 301.8 ± 9.4 g in Phase 1: 305.8 ± 5.1 g (IP delivery group) and 297.8 ± 11.6 g (SC delivery group). Mean rat BW was: 287.2 ± 9.0 g in Phase 2: 289.4 ± 12.0 g (AgNP group), 282.4 ± 4.7 g(AgNP‐P407 group) and 289.8 ± 8.5 g (P407 group). Total dose of AgNP/kg BW was 0.03 mg Ag/kg and total dose of P407/kg ranged from 3.3 to 6.7 mL/kg BW.

Mild redness, swelling, and scabbing were noted initially between days 3 to 5 post‐implantation in 14 rats. This included one rat in Phase 1 at the control site secondary to over grooming and 13 of 15 rats in Phase 2 after IP delivery. Of these 13 rats, three received AgNP (none of which exhibited swelling), five received AgNP‐P407 (3 of which exhibited swelling) and five received P407 (two of which exhibited swelling). No macroscopic lesions were present on any organ during necropsy on day 7 in any rat.

### 3.2 Blood analyses

**FIGURE 1:**
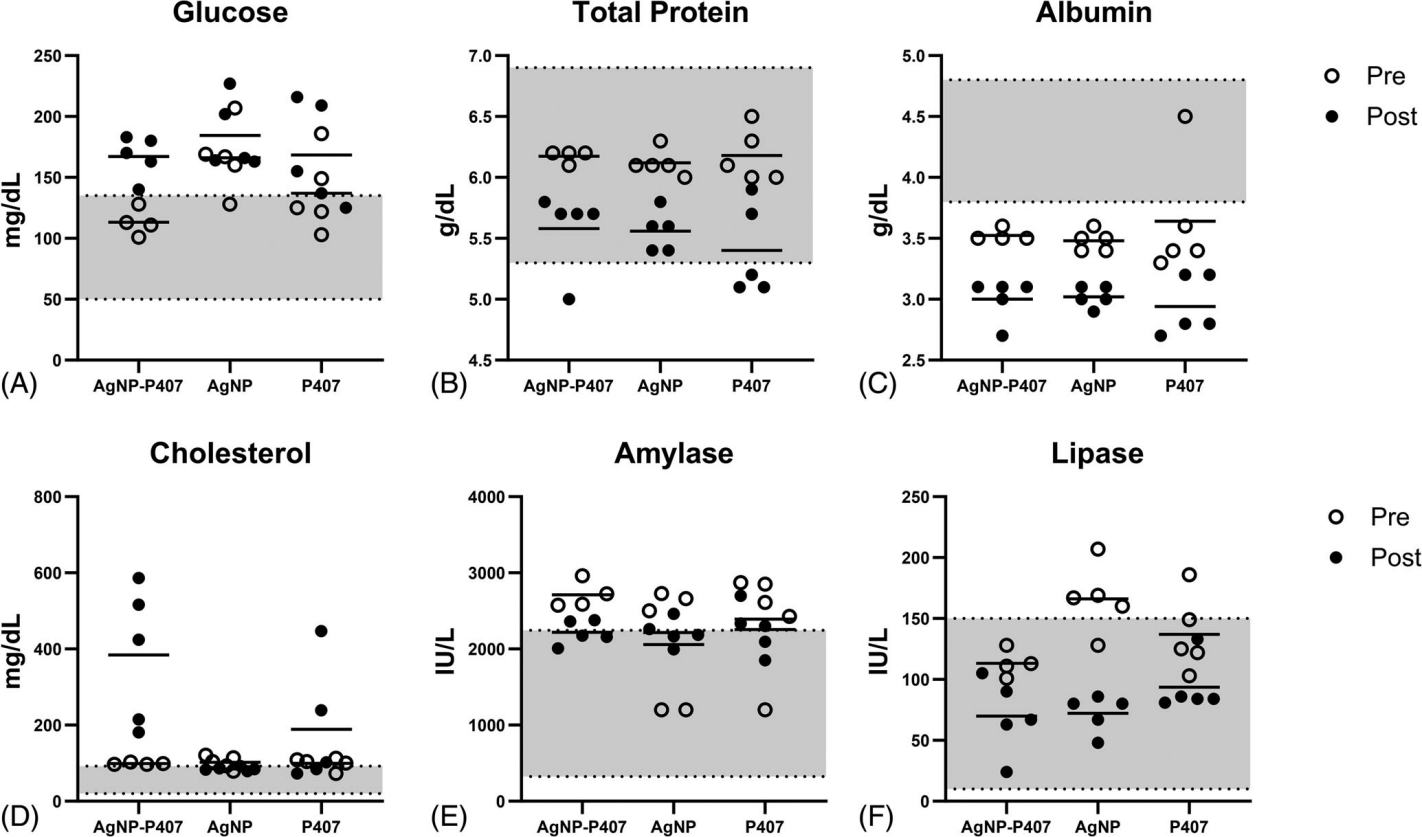
Biochemistry values for rats pre‐ and 7 days post‐application in Phase 2. Parameters that were outside of reference range in more than two individual rats are shown below, except for gamma glutamyl transferase (GGT), as that was reported as: <10 IU/L (reference 2, 3 IU/L) in all rats. The reference range is shaded gray. All rats are represented by individual dots (open dot = pre and solid dot = post). Glucose was increased whereas total protein, albumin, and lipase were decreased in rats from all groups compared to preoperatively. Cholesterol was increased in the groups that received poloxamer 407 (P407), but not in rats that only received AgNP.

Post‐study chemistry panels were available for all rats. Prestudy chemistry panels were available for 14/15 rats in Phase 2 (one rat in the AgNP‐P407 composition group had insufficient blood for analyses) and for none in Phase 1. Cholesterol was increased only in groups that received P407; values in Phase 2 rats differed from preoperative values in all groups (Phase 1 is represented in Table 2; Phase 2 is shown in Figure 1).

**TABLE 2:** Biochemistry for all rats in Phase 1 postoperatively.

Value	Ref range	AgNP‐P407 IP					AgNP‐P407 SC				
		Rat 1	Rat 2	Rat 3	Rat 4	Rat 5	Rat 1	Rat 2	Rat 3	Rat 4	Rat 5
Glu	50–135	124	130	108	**150**	123	117	111	122	133	135
Alb	3.8–4.8	**3.4**	**3.4**	**3.3**	**3.4**	**3.4**	**3.2**	**2.9**	**2.6**	**3.1**	**3.2**
GGT	2–3	**<10**	**<10**	**<10**	**<10**	**<10**	**<10**	**<10**	**<10**	**<10**	**<10**
Amylase	326–2246	**2293**	**2525**	**2454**	**2417**	**2258**	2071	2009	1757	2065	**2386**

*Note*: All values for any parameter that had a result outside of reference range in more than two rats are listed, with those outside of reference range bolded. All other parameters were within normal limits.

Abbreviations: Alb, albumin; GGT, gamma glutamyl transferase; Glu, glucose.

Post‐study CBCs were available for all rats in Phase 1, 9/15 rats in Phase 2 (3/5 rats that received AgNP; 5/5 rats that received AgNP‐P407; 1/5 rats that received P407) with insufficient blood available post chemistry in the remainder due to clotting of the sample. Platelets were decreased in one rat in Phase 2 (31 K/uL) that received AgNP‐P407 and had lipemic serum and were within reference range in the remainder (694–1412 K/uL).^35^ No other abnormalities were noted.

### 3.3 Quantitative silver analysis

#### 3.3.1 Delivery route (Phase 1)

**FIGURE 2:**
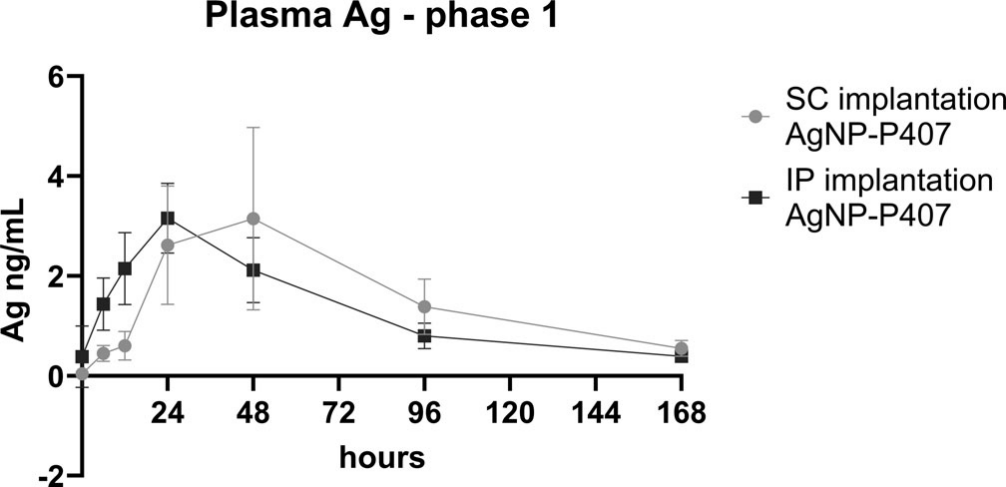
Plasma Silver (Ag) (ng/mL) in Phase 1. The plasma peak for intraperitoneal (IP) delivered AgNP in poloxamer 407 (“AgNP‐P407”) was earlier, with a peak at 24 h, whereas the peak for subcutaneous (SC) delivery was at 48 h.

**FIGURE 3:**
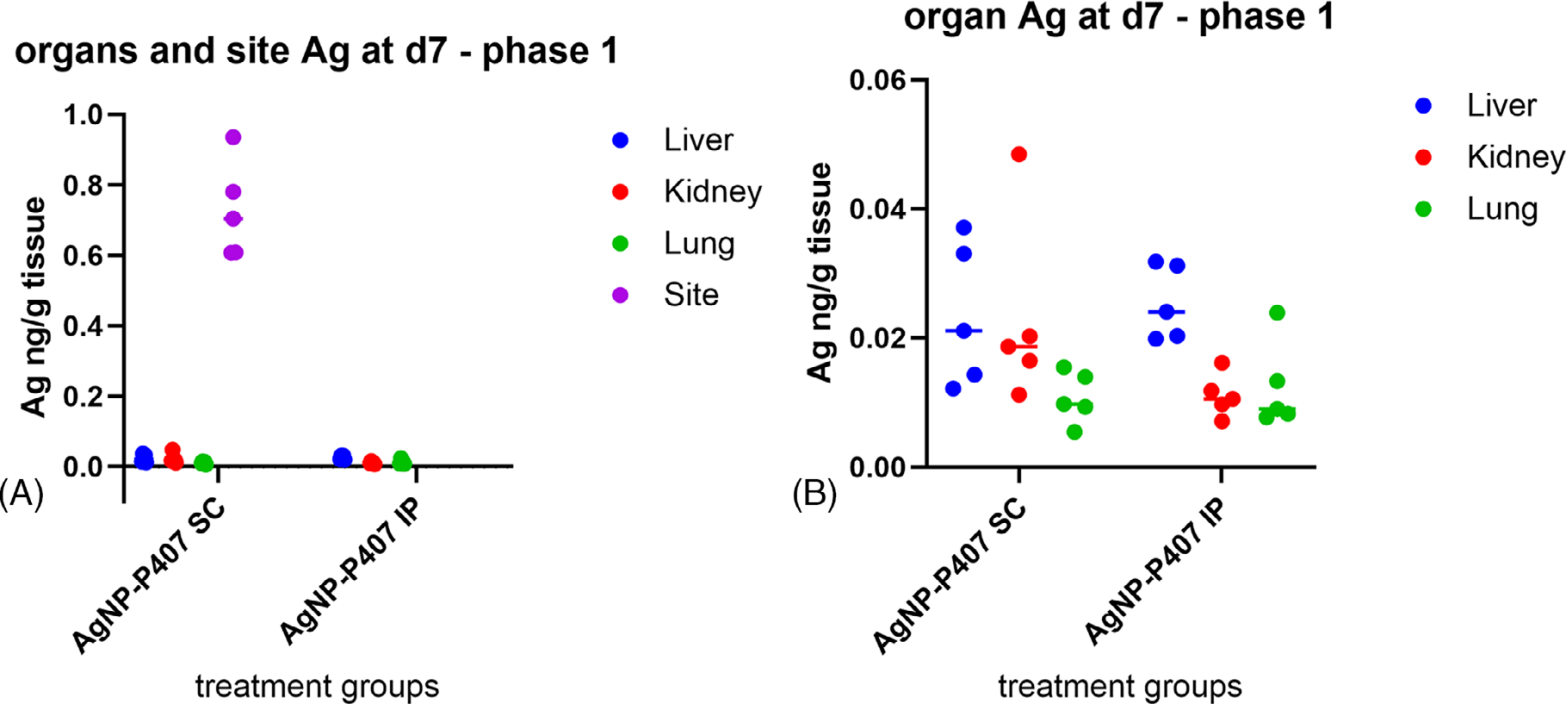
Silver (Ag) in organs and the subcutaneous (SC) delivery site (ng/g tissue) in Phase 1. (A) All samples, (B) organs only. The entire SC pocket in which the AgNP‐Po7 was delivered was excised and the Ag concentration in the SC implantation site remained higher at day 7 than the concentration found in any of the organs sampled. All values are represented as individual dots (*n* = 5 per group). The Ag found in liver was higher than in kidney or lung tissue, with this difference larger after intraperitoneal (IP) implantation.

The plasma Ag Cmax occurred at 24 h following IP delivery as compared to 48 h after SC delivery of AgNP‐P407; however, this difference was not statistically significant (*p* = .08; Figure 2). There was no difference in other noncompartmental parameters between the delivery sites during Phase 1 (Table 3). There was minimal Ag present in organs at day 7 after either IP or SC delivery (<0.0005% for all organs), although the liver contained more Ag than kidneys or lung (Figure 3). Silver content in the SC implantation site was higher than organ levels with values between 0.61–0.94 ng/g tissue (mean 0.73 ± 0.14 ng/g tissue, or 0.07% of AgNP delivered).

**TABLE 3:** Summary of the pharmacokinetic data.

		Phase 1						Phase 2					
		Subcutaneous (SC)			Intraperitoneal (IP)			IP AgNP			IP AgNP‐P407		
Parameter	Units	Median	Min	Max	Median	Min	Max	Median	Min	Max	Median	Min	Max
Cmax	ng/mL	3.35	1.22	5.90	3.20	2.23	3.93	1.37	1.15	1.72	1.56	0.61	48.00
Tmax	h	48.00	24.00	48.00	24.00	24.00	24.00	12.00	6.00	24.00	24.00	24.00	
AUClast	h*ng/mL	233.51	111.64	383.42	220.25	154.71	294.19	97.54	55.77	122.89	100.89	66.34	221.30
AUC0‐inf	h*ng/mL	263.16	156.43	426.25	242.62	177.60	340.20	136.26	126.60	310.01	164.86	87.95	281.94
AUC% extrap	%	12.21	10.05	28.64	11.72	7.68	13.52	34.63	22.95	62.13	24.57	20.83	50.58
Lamba z	1/h	0.014	0.009	0.017	0.014	0.013	0.016	0.008	0.003	0.009	0.011	0.005	0.012
T 1/2 lambda z	h	49.92	40.83	76.50	48.92	42.72	51.87	91.13	81.53	232.98	64.85	56.34	148.62
AUMClast	h*h.*ng/mL	15995.77	9097.67	26069.08	12192.94	8907.95	17284.14	6679.58	3893.14	9137.76	8368.17	4616.46	14408.54
MRTlast	h	68.50	66.93	81.49	58.43	55.36	60.08	69.81	62.04	77.84	69.59	64.29	82.94

#### 3.3.2 Delivery composition (Phase 2)

**FIGURE 4:**
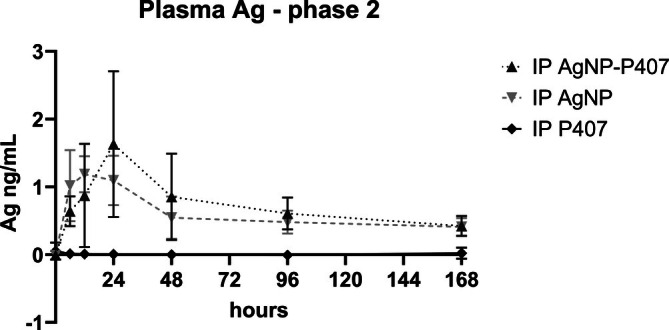
Plasma silver (Ag) (ng/mL) in Phase 2. The full plasma sample rather than a standardized volume was diluted and digested for analysis. While the resultant data provides an indication of timing of the peak plasma concentration between the three administration formulations, all delivered intraperitoneally (IP), the graph is only provided to show the difference in time due to the potential variation between samples. The plasma peak for non‐mixed AgNP was earlier and flatter than the peak for the sustained release formulation (“AgNP‐P407”). No peak or uptake was seen for poloxamer 407 only (“P407”).

**FIGURE 5:**
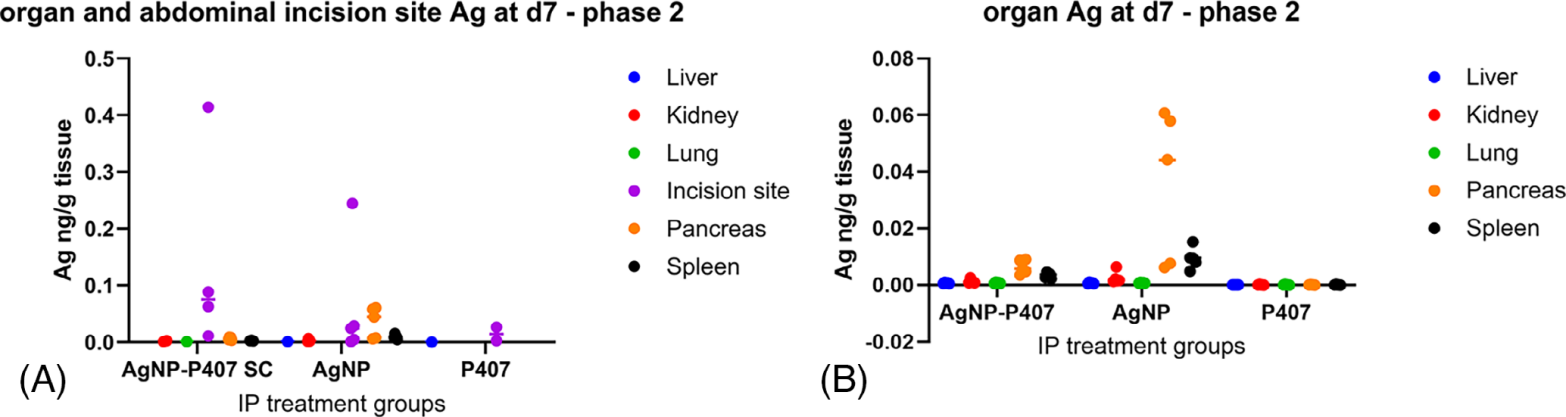
Silver (Ag, ng/g tissue) in organs and the abdominal incision in Phase 2. (A) All samples, (B) organs only. The abdominal incision used to implant the product intraperitoneally was also sampled. The Ag concentration in the incision was higher than the organ uptake, most likely due to local absorption during implantation. Interestingly, the amount of Ag in the pancreatic samples with non‐mixed AgNP was higher than for the other two groups. The silver found in the incision of the poloxamer 407 only group (“P407”) might have been due to contamination via instruments at time of surgery. (B) Pancreatic tissue in rats who received AgNP contained more Ag at necropsy than rats that received AgNP in P407.

The plasma Ag Cmax occurred earlier (*p* = .04) for AgNP delivered without P407 (Tmax = 12 h) than for AgNP delivered in P407 (Tmax = 24 h); however, no other non‐compartmental parameters were different between the two formulations (Table 3 and Figure 4). The mean Ag content of the pancreas in three rats who received AgNP in its own solution without P407 was 0.035 ng/g tissue (0.044; 0.058 and 0.060 ng/g tissue for 3 rats, and 0.006 and 0.007 ng/g tissue for the other 2 rats). This corresponded with 0.0001%–0.0006% of delivered Ag. The Ag content of the remainder of pancreatic tissues were comparable with Ag values of other organ tissue Ag, with several BLOQ (Figure 5). No other sampled organs contained appreciable levels of Ag. The mean Ag measured in tissue of the abdominal incision was 0.12 ± 0.17 ng/g tissue (<0.001 to 0.4 ng/g tissue) in the group that received AgNP‐P407, which was higher than after AgNP administration: 0.06 ± 0.1 ng/g tissue (0.001 to 0.24 ng/g tissue). The Ag measured in the abdominal incision was higher than the values found in any organs in four rats: three that received AgNP‐P407, and one that received AgNP without P407. Silver also was found in the incision site of rats who did not receive AgNP, but only at a level comparable to organs in the same group (0.006 ± 0.01 ng/g tissue).

### 3.4 Histologic assessment

**FIGURE 6:**
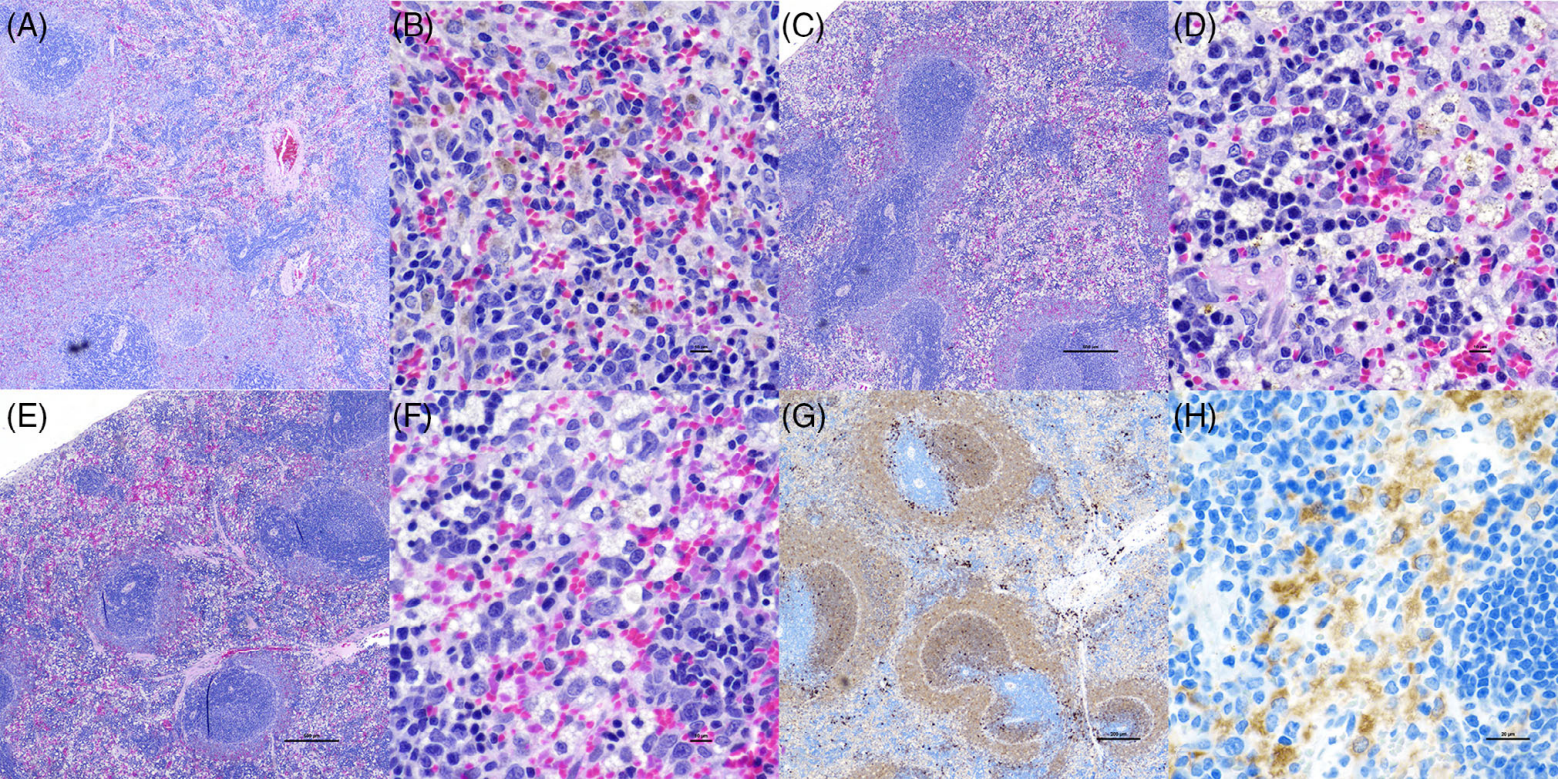
Photomicrographs of a hematoxylin and eosin (H&E) stain of the spleen in three different rats from Phase 2 at 20× magnification (A, C, E) and 400× magnification (B, D, F), and CD68 labeling at 20x magnification (G) and 400× magnification (H). Group allocation of IP surgical delivery was as follows: (A, B) Rat that received AgNP. (C, D) Rat that received AgNP‐P407. (E, F) Rat that received P407. (G, H) Rat that received P407. Abundant macrophage vacuolization is seen in the two rats that received P407 (C, D and E, F) with little distinction between the rat that received AgNP‐P407 and the rat that only received P407. The CD68 positively identified the vacuolated cells as macrophages.

The spleen consistently showed histopathologic changes characterized by macrophage hyperplasia with vacuolation and aggregation (Tables 4 and 5), more abundantly so in rats receiving P407 (Figure 6). Histopathologic changes were noted in all incisions and implantation sites where an incision was noted. In one rat in Phase 2 no lesion was identified in the abdominal area that was sampled to capture the abdominal incision.(Tables 6 and 7). The control SC implantation site with P407 had more fibrosis than the corresponding implantation site (*p* = .019; Table 6).

**TABLE 4:** Histopathology results for organs of rats in Phase 1.

	Heart	Kidneys	Liver	Spleen	Lung
IP‐1	ndl	ndl	ndl	Aggregates of macrophage hyperplasia with vacuolation	ndl
IP‐2	ndl	ndl	ndl	Aggregates of macrophage hyperplasia with vacuolation	ndl
IP‐3	ndl	ndl	ndl	Aggregates of macrophage hyperplasia with vacuolation	ndl
IP‐4	ndl	ndl	ndl	Aggregates of macrophage hyperplasia with vacuolation	ndl
IP‐5	ndl	ndl	ndl	Aggregates of macrophage hyperplasia with vacuolation	ndl
SC‐1	ndl	ndl	ndl	Aggregates of macrophage hyperplasia with vacuolation	ndl
SC‐2	ndl	ndl	ndl	Aggregates of macrophage hyperplasia with vacuolation	ndl
SC‐3	ndl	ndl	ndl	Aggregates of macrophage hyperplasia with vacuolation	ndl
SC‐4	ndl	ndl	ndl	Aggregates of macrophage hyperplasia with vacuolation	ndl
SC‐5	ndl	ndl	ndl	Aggregates of macrophage hyperplasia with vacuolation	ndl

*Note*: Splenic samples consistently showed changes. Macrophages form aggregates when they cannot completely degrade ingested macromolecules. Skin samples were graded and scored separately.

Abbreviations: n/a, organ not assessed/available; ndl, no diagnostic lesions found. “IP”, rats that received AgNP‐P407 intraperitoneally, “SC”, rats that received AgNP‐P407 subcutaneously.

**TABLE 5:** Histopathology results for organs of rats in Phase 2.

	Heart	Kidneys	Liver	Spleen	Lung	Thymus	Pancreas	Duodenum	LN
P407‐1	ndl	ndl	ndl	Macrophage hyperplasia with vacuolation	ndl	ndl	ndl	ndl	Macrophage hyperplasia with vacuolation
P407‐2	ndl	ndl	ndl	Macrophage hyperplasia with vacuolation	ndl	ndl	n/a	n/a	n/a
P407‐3	ndl	ndl	ndl	Macrophage hyperplasia with vacuolation	ndl	ndl	Increased cells in interstitium	ndl	Macrophage hyperplasia with vacuolation
P407‐4	ndl	ndl	ndl	Macrophage hyperplasia with vacuolation	ndl	ndl	ndl	n/a	n/a
P407‐5	ndl	ndl	ndl	Macrophage hyperplasia with vacuolation	ndl	ndl	ndl	n/a	Macrophage hyperplasia with vacuolation
AgNP‐P407‐1	ndl	ndl	ndl	Macrophage hyperplasia with vacuolation	ndl	ndl	ndl	n/a	n/a
AgNP‐P407‐2	ndl	ndl	ndl	Macrophage hyperplasia with vacuolation	ndl	ndl	ndl	n/a	n/a
AgNP‐P407‐3	ndl	Increased mononuclear cells	ndl	Macrophage hyperplasia with vacuolation	ndl	ndl	Increased mononuclear cells	n/a	n/a
AgNP‐P407‐4	ndl	ndl	ndl	Macrophage hyperplasia with vacuolation	ndl	ndl	ndl	n/a	n/a
AgNP‐P407‐5	ndl	ndl	ndl	Macrophage hyperplasia with vacuolation	ndl	ndl	ndl	ndl	Macrophage hyperplasia with vacuolation
AgNP‐1	ndl	ndl	ndl	Diffuse macrophage vacuolation, but less abundant	ndl	ndl	ndl	n/a	n/a
AgNP‐2	ndl	ndl	ndl	Diffuse macrophage vacuolation, but less abundant	ndl	ndl	ndl	n/a	n/a
AgNP‐3	ndl	ndl	ndl	Diffuse macrophage vacuolation, but less abundant	ndl	ndl	ndl	n/a	n/a
AgNP‐4	ndl	ndl	ndl	Diffuse macrophage vacuolation, but less abundant	ndl	ndl	ndl	n/a	Diffuse macrophage vacuolation, but less abundant
AgNP‐5	ndl	ndl	ndl	Diffuse macrophage vacuolation, but less abundant	Areas of hemorrhage	ndl	ndl	n/a	n/a

*Note*: Splenic and LN samples consistently showed changes.

Abbreviations: na, organ not assessed/available; ndl, no diagnostic lesions found; LN, lymph node. “P407”, rats receiving poloxamer 407 only, “AgNP‐P407”, rats receiving AgNP in poloxamer 407; “AgNP”, rats receiving AgNP only.

**TABLE 6:** Histopathology scores for the subcutaneous incision of rats in Phase 1.

	Inflammation	Necrosis	Edema	Hemorrhage	Fibrin	Fibrosis	Sum
Delivery site‐1	3	2	1	2	0	0	8
Delivery site‐2	3	1	0	1	0	0	5
Delivery site‐3	3	1	1	0	0	0	5
Delivery site‐4	4	3	2	0	0	0	9
Delivery site‐5	3	1	1	0	0	0	5
Mean	3.2	1.6	1	0.6	0	0	6.4
Median							
Sham‐1	3	1	1	1	0	1	7
Sham‐2	3	2	1	0	0	1	7
Sham‐3	3	1	1	0	0	1	6
Sham‐4	3	1	1	0	0	0	5
Sham‐5	3	1	0	0	0	1	5
Mean	3	1.2	0.8	0.2	0	0.8	6
Median							

*Note*: Lesions (inflammation and leukocyte types, necrosis, edema, hemorrhage, fibrin, fibrosis) and their severity were semi‐quantitatively scored as follows: 0 = no lesions; 1 = minimal; 2 = mild; 3 = moderate; 4 = marked, with a total score provided (“sum”). A Mann Whitney U test was performed (www.statskingdom.com).

**TABLE 7:** Histopathology scores for the abdominal incision of rats in Phase 2.

	Inflammation	Necrosis	Edema	Hemorrhage	Fibrin	Fibrosis	Sum	Comments
P407‐1	3	3	2	0	0	2	10	
P407‐2	2	1	0	0	0	2	5	
P407‐3	3	2	1	0	0	2	8	
P407‐4	3	3	1	0	0	2	9	
P407‐5	4	4	2	0	3	2	15	Abscess with suture noted
AgNP‐P407‐1	3	1	2	0	0	1	7	
AgNP‐P407‐2	2	2	1	0	0	2	7	
AgNP‐P407‐3	2	2	1	0	0	1	6	
AgNP‐P407‐4	2	2	0	0	0	2	6	
AgNP‐P407‐5	3	2	0	0	0	1	6	
AgNP‐1	4	4	1	0	0	1	10	
AgNP‐2	2	1	0	0	0	2	5	
AgNP‐3	3	1	2	0	0	2	8	
AgNP‐4	2	1	0	0	0	2	5	
AgNP‐5	0	0	0	0	0	0	0	No lesion/incision seen

*Note*: Lesions (inflammation and leukocyte types, necrosis, edema, hemorrhage, fibrin, fibrosis) and their severity were semi‐quantitatively scored as follows: 0 = no lesions; 1 = minimal; 2 = mild; 3 = moderate; 4 = marked, with a total score provided (“sum”). “P407”, rats receiving poloxamer 407 only, “AgNP‐P407”, rats receiving AgNP in poloxamer 407; “AgNP”, rats receiving AgNP only.

## 4 DISCUSSION

Delivery of AgNP‐P407 SC or IP did not lead to any clinically noted adverse reactions. A plasma Ag peak was seen after both IP and SC delivery, while delivery in P407 delayed the systemic uptake. Silver was retained at the SC delivery site at 7 days post‐implantation without adverse effects noticed locally or histologically. The amount of Ag present in organs at 7 days post‐delivery was minimal for either delivery route.

Bloodwork changes were present at 7 days postoperatively in all Phase 2 rats. Some changes, such as the decrease in total protein and albumin most likely were secondary to undergoing surgery, while the increase in glucose could be stress related. An increase in cholesterol was seen in rats receiving P407. This has been described previously in rats^36^ but has not been reported in other animals. This could either be species dependent or might be due to the relatively high volume of P407 to BW ratio in rats as opposed to dogs or cats.

Acute toxicity of silver administered at high doses has been described, with a reported fatal dose of 50 mg colloidal Ag IV,^37^ or 10 g elemental silver.^38^ In rats, crossing of the blood brain barrier was described after SC injection of 62.8 mg/kg AgNP,^39^ while a different study looking at lower doses found that a biomedical application at <10 mg/kg AgNPs was safe but a dose >20 mg/kg was not.^40^ Systemic absorption and organ uptake was demonstrated to be higher for smaller size particles (10 nm) than 40 nm or 100 nm particles in mice.^41^ Histologically, the tissues at the SC implantation site showed only minor inflammation and fibrosis. Necrosis of hepatocytes as well as infiltration of inflammatory cells have been described after repeated IV delivery in mice.^42^ The mice were administered 35 IP injections of 2 mg/kg AgNP of the same size and stock citrate buffered solution as used in this study. The dose delivered in our study was lower, and it was a single dose, which might explain the lack of toxic changes to the organs. Local migration, inflammation and necrosis were seen after high dose (0.1 g/kg) intravaginal delivery in rabbits.^43^ In our study, no evidence of consistent changes to any of the organs was seen, except for changes to the spleen.

Vacuolization of splenic macrophages was seen in all rats in Phase 1, and this vacuolization was identified to correspond with P407, rather than secondary to AgNP administration in Phase 2. Macrophages form aggregates when they cannot completely degrade ingested macromolecules, most likely parts of P407 or cholesterol secondary to the hypercholesterolemia found in the rats. This finding might limit the dose and ability for repeat administrations, especially of high doses of P407, in smaller animals. The rats in this study received approximately 3.6 m LP407/kg BW, which would be substantially higher than any of the volume administered in clinical veterinary patients. The high volume to BW ratio in rats might also further explain the finding of hypercholesterolemia in our study and hyperlipidemia described previously in rats,^44^ mice^45^, ^46^ and rabbits^47^, ^48^ in other studies but not reported in other species. A significant increase in serum cholesterol and triglicerides (TGs) was seen 24 h and 5 days after the last administration of a 30‐day, dosing protocol of 300 mg/kg P407 IP twice weekly.^45^ When this protocol was repeated, with blood levels measured at 24 h, 4 days and 10 days, serum cholesterol returned to within normal limits (wnl) on day 4 and 10, while TGs were still elevated on day 4 but wnl on day 10, indicating that the increase was temporary and self‐limiting.^46^ Potentially the length of the increase could be aligned with the duration of administration. Serum TGs and cholesterol was elevated on day 2 in rabbits administered 137.5 mg/kg 22% P407 (0.625 mL/kg) SC, but not at 5.5 or 27.5 mg/kg.^47^ No elevation was seen at 6 h, day 1, and levels returned to wnl on days 7 and 14, indicating both a delayed uptake and self‐limiting effect. When these doses were given IV in an ear vein, and serum TGs and cholesterol measured at 2, 4 and 7 days, a significant increase in serum TGs was seen at 2 days, with values wnl at days 4 and 7.^48^ Cholesterol was increased at day 2 (not significantly), and wnl days 4 and 7. Given that no earlier measurements were performed, it is possible that an earlier and more pronounced increase in both was missed.

For Phase 1, the plasma Ag Tmax and Cmax were similar between the two delivery locations (12 h for IP and 24 h for SC), the plasma Ag levels rose more slowly and with more variability compared to the IP delivery, although plasma Ag levels were similar at day 7 between the two sites. This finding was further corroborated by the presence of Ag in local subcutaneous tissues on day 7. Only AgNP‐P407 was subcutaneously implanted, so a comparison of uptake with or without P407 cannot be made. However, both compositions were delivered IP allowing a comparison. Plasma Ag levels for AgNP‐P407 peaked at 24 h, compared to a peak at 12 h for AgNP only, further substantiating the prolonged delivery and delayed absorption of AgNP delivered in P407. This was similar to delivery of other agents in P407, such as carboplatin, where plasma platinum (Pt) was highest at 24 h (first post‐delivery sample), and local samples had a 6.7 times higher Pt value than plasma at day 7.^22^ Blood sampling times were focused on the earlier post‐delivery times rather than later in the study, based on prior documented plasma peaks and uptake of drugs delivered subcutaneously in P407 in rats.^22^ While ideally more samples per rat would have been acquired, the number was restricted for animal welfare purposes, given the blood volume collected at each time point.

While the liver contained more Ag than the kidneys or other organs, organ distribution at 7 days post‐delivery was minimal for all delivery routes and delivery compositions, except for the incision itself and for the pancreas in the rats that received AgNP IP. However, care should be taken when comparing tissue concentration to plasma levels, especially in the context of efficacy and activity. Homogenates of tissue samples represent both the intracellular (due to cell lysis) and extra cellular compartments and do therefore not necessarily reflect the active component of a drug.^49^ The abdominal wall incision sites of rats that received AgNP or AgNP‐P407 IP contained Ag. For four rats (3 that received AgNP‐P407, and 1 that received AgNP) this value was substantially higher than other tissue values, and most likely due to AgNP being captured in the tissues of the abdominal incision at time of surgical intraperitoneal delivery. A higher amount of Ag than baseline was present than in the incision of two of rats that only received P407. This most likely was due to contamination via instruments at the time of surgery rather than during necropsy as none of the other samples of these two rats showed the same pattern. While instruments were cleaned and resterilized with a bead sterilizer, it is possible that not all AgNP was fully removed between rats. This highlights the risk of contamination and environmental risk while handling AgNP. Consideration should be given to disposable instruments used for the delivery of AgNP as well as double‐gloving. This would minimize exposing personnel or other patients (in case of surgical instruments) to AgNP. As the higher level of pancreatic tissue Ag was only seen in rats that received AgNP, it might be possible that Ag was directly absorbed into the pancreatic tissue, as opposed into other organs that have a more defined capsule such as liver, kidney and spleen. The delivery of AgNP in P407 might have decreased the direct exposure of pancreatic tissue to Ag and therefore absorption.

While hypercholesterolemia was seen in rats receiving P407, administering AgNPs in P407 did not cause organ or local toxicity. The hypercholesterolemia also might be the underlying etiology for the vacuolization in splenic and lymph node samples, although AgNP itself also might be a contributing factor. Still, the lack of increased Ag in splenic tissue would not support AgNP to be the driving factor for these changes. The rats that developed hyperlipidemia in an earlier study^44^ did not develop similar lesions in the spleen. However, the P407 they received was 10% (w/w) whereas the rats in our study received 30% w/w, which might account for the differences observed. Vacuolization of macrophages has been described previously in livers of mice after chronic administration (300 mg/kg twice weekly for 30 days).^45^ However, changes in splenic weight, cell number and population were noted after the same protocol in a different study.^46^

There was more individual variability in plasma Ag concentrations in Phase 2 for both formulations, which led to an unacceptably larger percent extrapolations of the area‐under‐the‐curve to infinity (>25% extrapolated for 2/5 rats in the AgNP P407 and 4/5 rats in the AgNP group). This led to a longer unreliable estimation of the terminal phase for these animals. While this data was reported in this manuscript, it should only be used as a basis for any future studies since the half‐life is not accurately estimated and is considered a limitation of this analysis. Due to dilution of the full sample as opposed to dilution of specific volume of sample for the plasma Ag analysis in Phase 2 there was inconsistency in the dilution factor for this analysis. These results should therefore only be considered as an approximation and were included to show trends& time to plasma peak. Consideration was given to repeating the full experiment, measurement of markers within the plasma to calculate dilution, but not all plasma samples were available for reanalysis. Lastly, omission of all the Phase 2 Ag data was considered; however, the authors felt that adding the data was worthwhile, as it would still show trends of time until plasma peak between the three groups with a different drug delivery composition. The time of the plasma peak of AgNP‐P407 IP in Phase 2 matched the peak in Phase 1 with the shape of the curve matching. Given the sampling method with a specified amount of blood (0.3 mL of blood for all pre‐euthanasia samples), the variation in plasma volume was likely small for the live animal samples, and all shown data was a mean from five rats. Another limitation was that the application of AgNP‐P407 was performed in non‐infected environments and a surgically created wound, and uptake in inflamed or infected wounds with secondary changes to local tissues and vasculature might differ. In addition, it is possible that the P407‐induced hyperlipidemia in rats affected drug distribution and elimination. Care should therefore be taken with extrapolating results to other species.

No local or systemic adverse effects were seen after local delivery of AgNP‐P407 in rats, and no tissue toxicosis was noted 7 days after delivery. Delivery in P407 delayed systemic uptake of Ag after IP delivery of AgNP. Direct absorption into organs without a clear fibrous capsule or an incisional approach might occur. Local delivery and use of AgNP‐P407 in contaminated wounds or infected locations may be possible for topical treatment with prolonged local presence of Ag while having little systemic effects. However, further testing on efficacy and dosing is needed.

## AUTHOR CONTRIBUTIONS

Risselada M, DVM, PhD, DECVS, DACVS (Small Animal): Contributed to the concept and design of the study, wrote the grant to obtain funding, participated in data collection, performed data analysis and wrote the manuscript. Anderson ML, DVM, DACVPM, Bates MG, BVSc, DACVS (Small Animal), Cox A, DVM, PhD, DACVP and McCain R, BS, RLATg: Contributed to the design of the study and the grant proposal, performed data collection, participated in data analysis, and assisted in writing the manuscript. Messenger K, DVM, PhD, DACVCP: Performed the pharmacokinetic analyses, assisting in writing the revised manuscript, and drafted the pertinent responses.

## FUNDING INFORMATION

This work was funded by a 2023 ACVS Foundation Diplomate Research Grant.

## CONFLICT OF INTEREST STATEMENT

Dr Risselada is an Associate Editor of Veterinary Surgery. She was excluded from the editorial and review process of this article. No AI‐assisted technologies were used in the generation of this manuscript.
